# Field testing of a prototype mechanical dry toilet flush

**DOI:** 10.1016/j.scitotenv.2019.02.220

**Published:** 2019-06-10

**Authors:** Jan Hennigs, Kristin T. Ravndal, Thubelihle Blose, Anju Toolaram, Rebecca C. Sindall, Dani Barrington, Matt Collins, Bhavin Engineer, Athanasios J. Kolios, Ewan McAdam, Alison Parker, Leon Williams, Sean Tyrrel

**Affiliations:** a School of Water, Energy and Environment, Cranfield University, United Kingdom; b Pollution Research Group, University of KwaZulu-Natal, South Africa

**Keywords:** BSC, Bristol Stool Chart, NMT, Nano Membrane Toilet, PRG, Pollution Research Group, SMF, Synthetic Menstrual Fluid, SOB, Silicone Rubber with oil-bleed effect, SU, synthetic urine, UDDT, Urine Diversion Dehydration Toilet, UKZN, University of KwaZulu-Natal, USFDA, United States Food and Drug Administration, VIP, ventilated improved pit latrine, WASH, Reinvent the toilet challenge, Iterative design, User testing, Science-design Interface

## Abstract

A prototype of a non-fluid based mechanical toilet flush was tested in a semi-public, institutional setting and in selected peri-urban households in eThekwini municipality, Republic of South Africa. The mechanism's functionality and users' perception of the flush were assessed. User perception varied depending on background: Users accustomed to porcelain water flush toilets were open to, yet reserved about the idea of using a waterless flush in their homes. Those who commonly use Urine Diversion Dehydration Toilets were far more receptive. The user-centred field trials were complemented by a controlled laboratory experiment, using synthetic urine, -faeces, and -menstrual blood, to systematically assess the efficiency of three swipe materials to clean the rotating bowl of the flush. A silicone rubber with oil-bleed-effect was found to be the best performing material for the swipe. Lubrication of the bowl prior to use further reduced fouling. A mechanical waterless flush that does not require consumables, like plastic wrappers, is a novelty and could – implemented in existing dry toilet systems – improve acceptance and thus the success of waterless sanitation.

## Highlights

• Prototype mechanical toilet flush successfully user-tested in households and lab
• User feedback positive but dependent on toilet habit
• Removal of fouling from soft faeces not satisfactory
• Surface lubrication improves fouling removal.

## Graphical abstract

**Figure:**
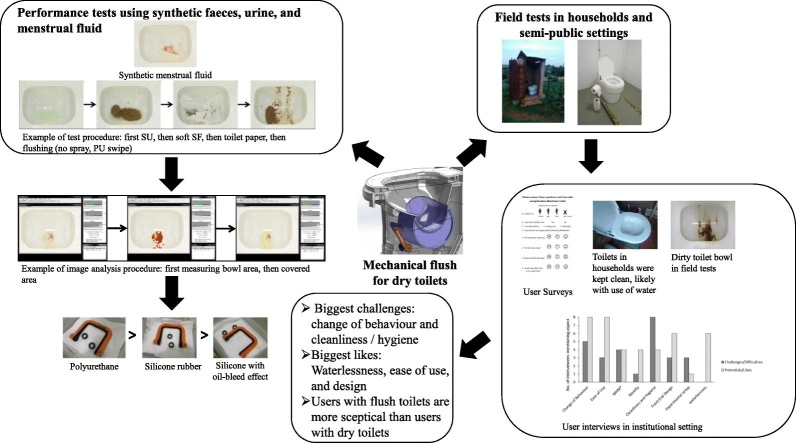

## 1 Introduction

4.5 billion people worldwide lacked access to safely managed sanitation in 2015, of which 892 million practiced open defecation. Safely managed sanitation is defined as “an improved sanitation facility that is not shared with other households, and where excreta are disposed of in situ or transported and treated off-site” (WHO and UNICEF, 2017). The entailing problems, such as groundwater pollution (Graham and Polizzotto, 2013), the transfer of pathogens and the resulting diseases (Curtis et al., 2000; Wolf et al., 2014), children (especially girls) missing school (Sclar et al., 2017), and increased risk of assault and rape, mainly for women when they have to urinate/defecate in the open (Jadhav et al., 2016; Miiro et al., 2018), are all serious inhibitors of human development (Jahan, 2016).

Providing sanitation for low income- and informal settlements in fast-growing cities of the Global South poses particular challenges: while the population in these areas is increasing, access to piped water and sewers is often limited (Cobbinah and Poku-Boansi, 2018; Parnell et al., 2007). Pit latrines and Urine Diversion Dehydration Toilets (UDDT) are common waterless sanitation solutions (Semiyaga et al., 2015). However, their pedestals (where present) can be considered uncomfortable, prone to fouling and malodour, and sometimes dangerous for children to use, as they can fall into the pit (Mkhize et al., 2017; Roma et al., 2013). This is a considerable obstacle for the acceptance of waterless sanitation technologies: if users prefer defecating in the open to using dry toilets, the provision of these toilets has little impact.

A solution could be a waterless toilet flush. Such a flush, as ‘user interface’, would be the physical barrier between the user and the faecal material inside the toilet, and thus have the potential to resolve problems of odour, of unpleasant visual contact with faeces and fouling, and of the danger of children falling into the pit. There currently are marketed applications of dry toilet flushes which seal the faeces in consumable materials, like the Dry Flush©, designed for camper vans or cottages not connected to sewers (Livingston and Roczynski, 2014) or the Loowatt toilet, already in use at British music festivals and in Madagascar (Natt et al., 2011), and technologies using small amounts of water, for example vacuum toilets (Wendland et al., 2007), or so-called microflush toilets (e.g. Mecca et al., 2013). There are also attempts to reduce water usage by reusing grey wastewater for toilet flushing (Garzon and Paterlini, 2018).

However, all of these technologies have problems with applicability to common dry toilet technologies used in cities of the global south: They either rely on at least some infrastructure for water, electricity, or both, or rely on specific consumables, such as plastic wrappers to contain the faeces. There currently are no published patents or research articles on purely mechanical, non-fluid based flush systems. This may be because a mechanical flush requires further storage or treatment of excreta on site, whereas water allows transport of the faecal material to a centralised treatment facility. Secondly, the development of a waterless flush is a challenging task, as faeces can be highly adhesive to various surfaces (Rose et al., 2015). Nonetheless, compared to alternative technologies, such a flush would have the environmental advantage that it does not consume water, electricity, or material in which to wrap the faeces. From a technical perspective, a mechanical flush is advantageous as it operates independently of outside infrastructure, like water or electricity. Its only requirement would be the manual operation by the toilet user, which would make its operational cost negligible, giving it yet another advantage over other available toilet flushes.

Another factor to consider is a flush's ability to convey solid waste, e.g. menstrual absorbents. While the disposal of solid waste into pit latrines and other sanitation systems is detrimental to transport and treatment of the faecal sludge, and is therefore discouraged, it frequently happens nonetheless. The reasons for this vary, but the lack of alternative solid waste disposal options and the stigma surrounding menstrual hygiene are two common examples (Radford et al., 2015; Tembo et al., 2017). A flush should not necessarily accommodate this behaviour, but it is likely that menstrual absorbents are disposed regardless of user instruction. Therefore, a certain level of resilience against these unwanted inputs should be achieved.

**Fig. 1:**
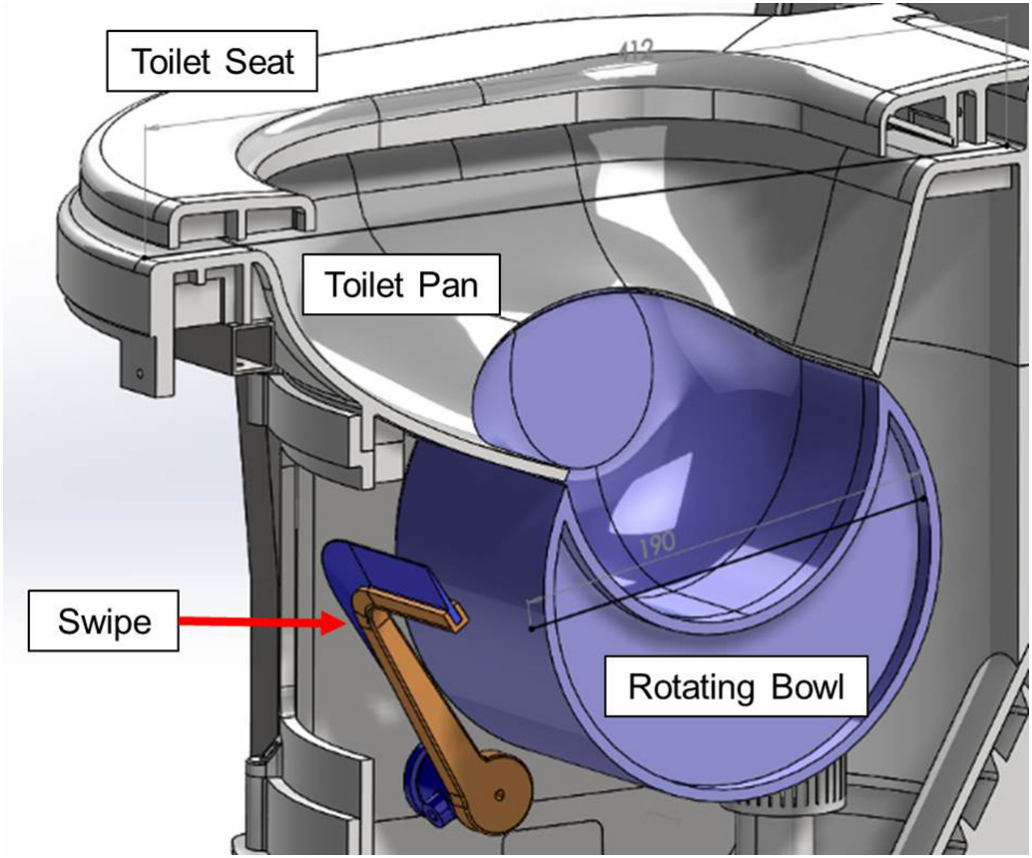
Cross section showing the rotating bowl and swipe – through gears connected to the toilet lid, the bowl rotates downward (from this perspective: counter-clockwise), and the swipe moves in concert to clean the bowl's surface.

To address these diverse challenges, based on an exploratory field study using household surveys in Kumasi, Ghana, Agile Innovation methods were employed to develop a mechanical flush which conveys faecal material from the toilet bowl and shields the users from the sight and odour of previous users' faecal matter (Tierney, 2017). A prototype of this mechanical flush was built to further its development by assessing its functionality and its reception by users (Fig. 1). Following the concept of user-centred design (Salah et al., 2015), the mechanical flush has to reliably provide satisfactory service, be perceived as clean, easy to use, and desirable, if it is to be successful.

Field trials are indispensable for the testing of prototypes in real-life scenarios (Ulrich and Eppinger, 2016) and in the spirit of Agile Innovation, prototypes should be tested on the product's target audience as soon as possible (Beaumont et al., 2017). However, the variability of frequency of toilet use and consistency of faeces (Rose et al., 2015) in authentic use conditions lead to a low level of control for the purpose of scientific performance evaluation. A trial large enough to eliminate these concerns would be prohibitively expensive. Therefore, the development of this novel technology will inevitably require controlled experiments to complement the findings from field tests. Both types of tests, the user-centred field trials and the controlled laboratory experiments, deliver valuable insights into the prototype's strengths and weaknesses. In combination, these findings are transferred into design recommendations, which form the basis for the next cycle of the iterative design process (Bresciani, 2015).

In exploration of the interface between design-based prototyping techniques and controlled experimental research, this paper presents the results of user-centred field testing and experimental evaluation of a mechanical toilet flush, which will inform the next design-iteration regarding user acceptance and swipe efficiency.

## 2 Methodology

### 2.1 Prototype description

**Fig. 2:**
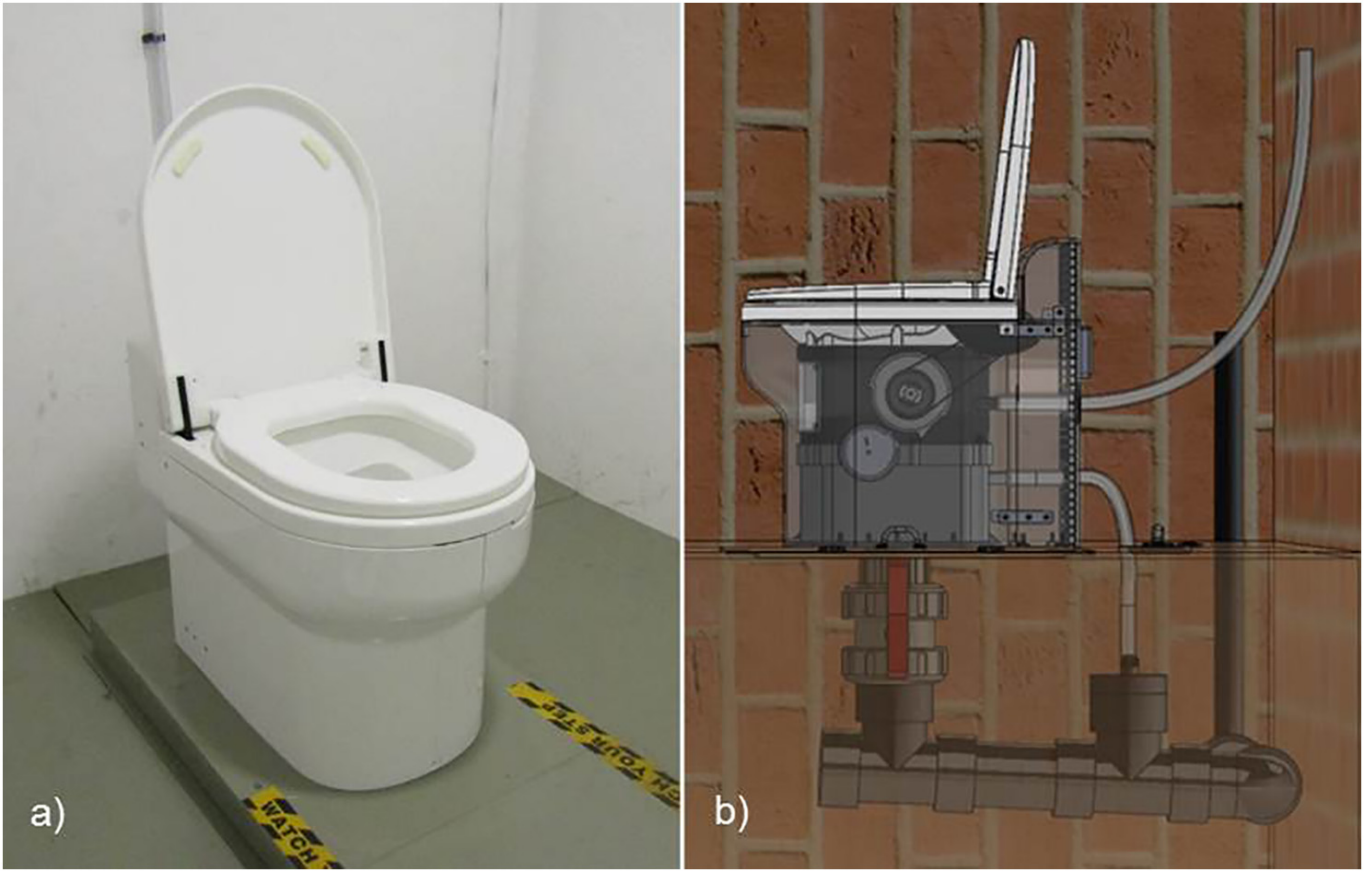
a) Prototype pedestal with mechanical waterless flush, installed in a dedicated toilet room adjacent to the laboratories of the Pollution Research Group at the University of KwaZulu-Natal; b) schematic of the installation: The pedestal is connected to the sewer mains and has a ventilation pipe from inside the unit. The gear system is shown to be on the side of the pedestal, underneath the cover.

The test object was a prototype pedestal incorporating the mechanical flush system, which is activated by moving the toilet's lid. Gears connect the lid to a rotating bowl that turns downward as the lid is closed. A swipe situated inside the pedestal is connected to the bowl-lid-gear-system. As the bowl rotates, the swipe moves downward, clearing remaining faeces out of the bowl. This mechanism acts as a barrier for visual and olfactory irritation of the user. Fig. 1 shows a cross section of the mechanical flush, with the rotating bowl and the swipe highlighted in blue and orange. The material used for the body of the prototype was polyurethane (*ALCHEMIX*® *VC 3341*; Alchemie Ltd., Warwick, England), a plastic often used in temporary sanitation systems, i.e. chemical toilets. A smooth, white surface finish resulted in an appearance similar to that of a porcelain water flush toilet, as can be seen in Fig. 2.

### 2.2 Study area

The inhabitants of eThekwini municipality, Republic of South Africa, represent a potential target customer group, as its peri-urban population is to a great extent not currently connected to sewered sanitation: in 2016/17, the municipality with a population of 3,820,174 provided 247,079 households with access to free basic level sanitation services, through UDDTs, existing ventilated improved pit latrines (VIP), or ablution blocks (eThekwini Municipality, 2017).

Hence, the prototype was tested in field trials in eThekwini. Firstly, one prototype unit was installed at the University of KwaZulu-Natal (UKZN) where it was situated in a designated toilet room adjacent to the laboratory facilities of the Pollution Research Group (PRG), and used by staff, students, and visitors for a total of 8 weeks. Fig. 2a) shows the prototype in the toilet room, and Fig. 2b) a schematic of the installation. Here, the unit was connected to a sewer pipe, which was flushed through with water on a regular basis to prevent a blockage of dry material.

Following the first month of testing at UKZN, two prototype pedestals (identical to the first) were successively tested in UDDT outhouses of six households in a low income community in a peri-urban region of the eThekwini municipality. The municipality supplies the water and sanitation services to the households, including construction, emptying, and maintenance of the UDDT facilities. Each household is supplied with 200 L of water per day, via a standpipe with a water restrictor, making water a limited resource too valuable to use for toilet flushing. The municipality, UKZN researchers and staff of Khanyisa Projects jointly identified households suitable and willing to participate in the study. Suitability was assessed on accessibility of the household, number of users of the toilet, and condition of the existing outhouse and pedestal. The prototypes replaced the UDDT-pedestals and flushed directly into the faecal sludge vaults underneath. At each household, the unit remained installed for one month.

### 2.3 Preliminary testing in a semi-public institutional setting at UKZN

Testing in an institutional setting at UKZN was considered the first real-use trial for the prototype, before it could be installed in households. Most importantly, the proper functionality of the flush had to be ensured, as the prototype would replace the households' only toilet pedestal. While the original pedestal remained with the families to be re-installed after the trial period, and in case the prototype malfunctioned, this would be an undesirable event for the families and was to be avoided.

### 2.4 Functionality tests

Based on its surface energy, Tierney (2017) identified silicone rubber as favourable material for the swipe and bowl-surface to maximise cleaning efficiency while reducing friction. He also assumed that a lubricated surface would be less prone to fouling than a dry surface, and proposed a surfactant solution for this aim. Based on these recommendations, and on availability in the manufacturing process, three different materials for the swipe were tested consecutively, namely *Poly PT Flex* **Polyurethane Rubber** (Polytek Development Corp., Easton, PA, USA), *Essil 291/292* **Silicone Rubber**, and *Essil 291/292* **Silicone Rubber with oil-bleed-effect (SOB)**, with the oil-bleed-effect creating a constant lubrication of the swipe's surface (Both Axson Technologies, Saint-Ouen-l'Aumône, France) (Table 1).

**Table 1:** Flush testing schedule at UKZN (test days are not counting weekends).

Swipe material	Polyurethane		Silicone		SOB	
Number of test days	6	7	6	7	6	7
Spray lubrication	No	Yes	No	Yes	No	Yes

The prototype production conditions required the rotating bowl to be of the same material as the pedestal itself (*ALCHEMIX*® *VC 3341 polyurethane*), which could not be alternated during the trials. Hence, to compare lubrication of the bowl's surface prior to use to the unlubricated surface, a pump-action spray bottle (as commonly used for disinfectant in laboratories and household toilets) filled with a solution of 10 g/L liquid hand soap in tap water was placed next to the pedestal and labelled “please give two sprays into the bowl before using the toilet” on 54% of trial days (Table 1). Thus, about 2 mL of soapy water were dispersed into the bowl before use of the toilet. To prevent users from cleaning fouling out of the bowl manually, no toilet brush or other cleaning equipment was supplied in the toilet room.

The data collected on swipe functionality were:

- Written observations about the handling and functionality of the prototype in a daily diary
- Photographs of the toilet bowl (daily) and the rubber swipes (upon removal)

As the input of faeces throughout a given day could not be determined, the effectiveness of the swipe was measured on a polar scale only, i.e. whether the photograph of the toilet bowl at the end of the day showed a clean bowl or not. After the daily photograph was taken, the bowl was cleaned.

### 2.5 Surveys and interviews

Surveys and interviews captured details of users' experience of, and attitude towards, using the prototype:

Short survey questionnaires were provided in the toilet room, and users were asked to fill one out every time they entered, even when they then decided against using the toilet. The intention was to gather information on the users' perception of the prototype's cleanliness, odour, ease of use and how they compared it to their usual toilet. A copy of the questionnaire used can be found in the Appendix A.

Eight of the users agreed to participate in semi-structured interviews about their experience using the toilet and their attitude towards it. The topics covered in the interview are listed in the Appendix B. All of the interviewees were either members of the PRG at UKZN or affiliated with it through their work, and so are well acquainted with sanitation research.

The interviews were conducted in English, recorded and transcribed. The transcripts were then coded to identify recurring themes of observations and attitudes of the users, as described by Weston et al. (2001).

Both the questionnaire and the interview topics were developed by the interdisciplinary author team of social scientists, designers and engineers. They were designed to gather information on the user experience in relation to the prototype, particularly those components which could be re-designed if users were dissatisfied. The questionnaire was reviewed by a social scientist of UKZNs School of Built Environment Development Studies who has experience working with communities in the peri-urban settlements of Durban, who confirmed that the questions being posed were both clear and culturally appropriate. The interview topics were reviewed by a Team Leader within PRG at UKZN who confirmed that members of the Pollution Research Group would understand and be open to answering them.

To further ensure that they were understood correctly, the questionnaires were publicly presented at the PRG offices at the beginning of the study and discussed informally during the trials. The interviews were held in a semi-structured manner specifically to ensure that the questions were understood correctly and that answers provided the information we needed.

### 2.6 Household tests

By replacing the UDDT pedestal, the prototype became the only available toilet for the household, and was thus used by all members of the household. Detailed instructions were provided by UKZN researchers and a representative of eThekwini municipality in person, and printed user instructions were also posted on the inside of the outhouse doors in isiZulu, the participants' native language. The families were provided cleaning equipment and asked to clean the prototype as they saw fit, but to use as little water as possible. Part of the instruction was to not use water when flushing the toilet.

After the initial tests at UKZN, the SOB swipe was considered to be the most promising with regard to cleaning effectiveness and resistance to fouling. Therefore, swipes of this material were used in the household units throughout the trials.

Analogous to the test at UKZN, the data collected during visits twice a week were:

- Written observations of the mechanism's functionality
- Photographs of the bowl's cleanliness
- Survey questionnaires in isiZulu

### 2.7 Swipe test in the laboratory

To further compare the three swipe materials: polyurethane, silicone, and SOB, they were tested in controlled laboratory experiments using:

- Simulant faeces in three consistencies, simulating solid, soft glutinous, and liquid faeces (type 3–4, 5–6, and 7 on the Bristol Stool Scale (BSC), respectively (Lewis and Heaton, 1997), see Appendix C)
- Synthetic urine as a secondary lubricant for the toilet bowl
- Toilet paper
- Tampons saturated with synthetic menstrual fluid
- The influence of lubricating the bowl's surface was tested by spraying a solution of 10 g/L liquid hand soap in tap water into the bowl before adding the simulant loads

A modified version of the mechanical flush prototype was used, which included the toilet lid, -seat, and flush mechanism, but had no connection to a sewer or faecal sludge vault. Instead, the simulant loads were flushed directly into a bucket underneath the rotating bowl. Photographs of the inside of the bowl were taken a) beforehand, b) after dropping in the simulant load, and c) after flushing the simulant load. This methodology was based on tests used during the development of the mechanical flush (Tierney, 2017).

**Fig. 3:**
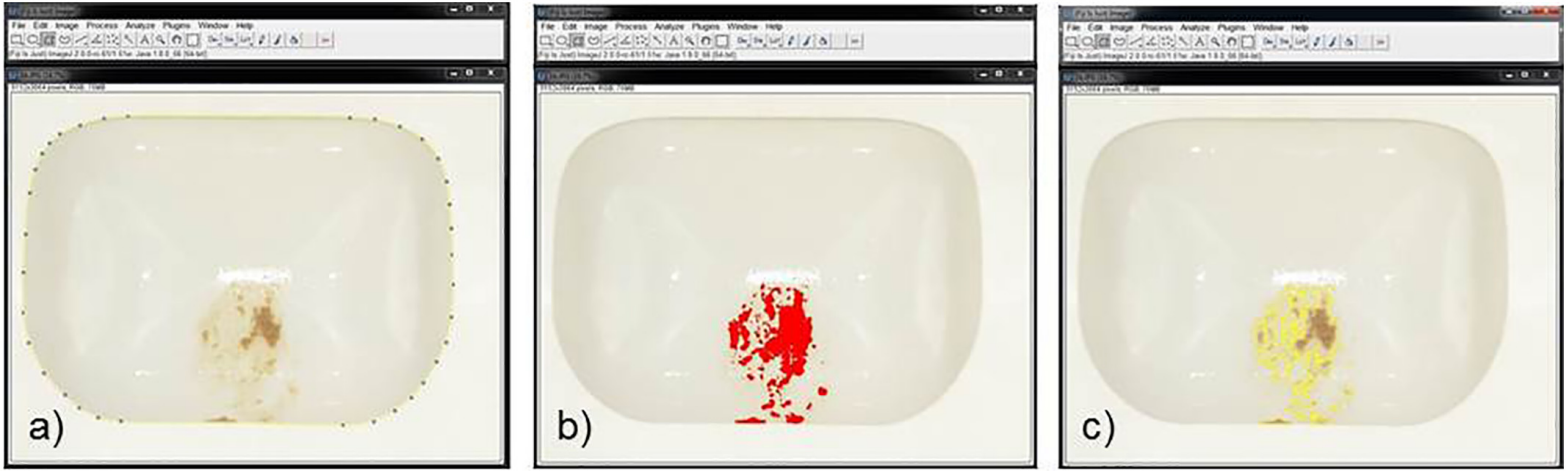
Image analysis using ImageJ software: a) with the *polygon selection* tool, the total area of the bowl is measured; b) with the *color threshold* tool, the fouled area was identified; c) shows the selection of the fouled area.

Using *ImageJ* software (FIJI-distribution, ImageJ development team at LOCI, University of Wisconsin-Madison) (Schindelin et al., 2015, Schindelin et al., 2012; Schneider et al., 2012), the fouled area relative to the area of the rotating bowl was determined before and after flushing (Fig. 3). Thus, a fouling-removal rate could be calculated:

(1) $$\mathrm{removal}\;\mathrm{rate}=1-\frac{{\left(\frac{A_{\mathrm{covered}}}{A_{\mathrm{bowl}}}\right)}_{\mathrm{After Flush}}}{{\left(\frac{A_{\mathrm{covered}}}{A_{\mathrm{bowl}}}\right)}_{\mathrm{Before Flush}}}$$

where A_covered_ is the area covered with faeces and A_bowl_ is the area of the bowl. Both were measured in pixels. Tests were conducted in triplicate.

#### 2.7.1 Simulant loads

For **synthetic faeces**, an adapted formula of the recipe proposed by Penn et al. (2018) was used. The recipe can be found in the Appendix D. Water content of 40%, 60% and 90% was used to simulate solid faeces of type 3–4, soft-glutinous faeces of type 5–6, and liquid faeces of type 7 on the BSC respectively. Harder faeces (types 1 and 2 on BSC) were not simulated, as the solid faeces already left no visible fouling.

**Urine** was simulated using an isotonic NaCl-solution (9 g_NaCl_/L) with 3 drops/L green food dye (“Extra Strong Green Food Colour Geld”, Dr Oetker UK Ltd., United Kingdom) in deionised water.

There is very little literature on **Synthetic Menstrual Fluid** (SMF), with the exception of medical research, e.g. (Christiaens et al., 1981; Dasharathy et al., 2012; Yang et al., 2012), nor are there many publications on the rheology or staining properties of real menstrual blood, which can partially be explained by its great variability in many parameters throughout the days of menstruation (Beller, 1971; Beller and Schweppe, 1979; Levin and Wagner, 1986), and partially by the apparent societal taboo to investigate or even talk about menstruation (Hertz, 2018; Spadaro et al., 2018; Wilson et al., 2018). In the tampon industry, the *syngina test* measures a tampon's absorbency (EDANA, 2015), in which *syngina fluid* is used. However, it has very little to do with the texture of actual menstrual fluid, but rather has a similar formula to the synthetic urine used in this study: The US-American Food and Drug Administration (USFDA), as well as the European Disposables and Nonwovens Association (EDANA), dictate the formula as “10 grams sodium chloride, 0.5 gram Certified Reagent Acid Fuchsin, 1,000 milliliters distilled water” (USFDA, 2010). Former member of the “tampon task force” (Vostral, 2017), Nancy Reame, cites an older FDA-recipe for syngina-fluid, pre-dating the standardisation of the syngina test, that seems to attempt to simulate a more realistic rheology of menstrual fluid: 10 g NaCl, 4 g NaHCO_3_, 4 g cellulose gum, 100 g glycerol, 880 g water, trace of food coloring (Marlowe et al., 1981 in: Reame, 1983). But while this liquid is thicker than the modern “syngina fluid”, it seems to have quite high surface tension and leaves no stains on the smooth, solid surface of the polyurethane toilet bowl tested for this study. From experience of the authors, real menstrual fluid is known to leave blood stains on porcelain toilet bowls, so this older formula could not be used either.

Instead, after some experimentation, a mixture of 40% defibrinated horse blood (TCS Biosciences Ltd., Botolph Claydon, United Kingdom), 10% glycerol (Mystic Moments UK, Fordingbridge, United Kingdom), and 50% isotonic NaCl-solution was prepared. An informal panel of colleagues who menstruate agreed that its viscosity, color, and staining properties sufficiently resembled those of real menstrual fluid. Medium absorbency tampons (Tampax® brand, Procter & Gamble, Weybridge United Kingdom) were fully saturated in this liquid, and then dropped into the toilet bowl from seat-level. This left a spatter pattern in the bowl. While tampons are far from being the most commonly used menstrual products in eThekwini municipality (Beksinska et al., 2015), and flushing of tampons, like other solid waste, should be discouraged for most sanitation systems, the method described above provided a useful way to administer blood stains to the toilet bowl as well as a test as to whether the mechanism could – in theory – handle flushing a tampon. Similarly, the flushing of menstrual pads was tested successfully, but this provided no useful staining of the toilet bowl and was thus not investigated further.

### 2.8 Ethics statement

The methodologies used for this study were approved by the Cranfield University Research Ethics Committee (all parts of the study, CURES approval numbers 3512, 3513, and 4534 for field work; 4750 for laboratory tests), and the Biomedical Research Ethics Committee at UKZN (the part of the study conducted in South Africa, i.e. the field testing, Approval Number BE409/17). Written, informed consent was obtained from all participants in either English or isiZulu, whereby the supplied information sheets emphasized the voluntary nature of participation and the option to leave the study at any time or opt out of answering any questions.

## 3 Results

### 3.1 Tests at UKZN

#### 3.1.1 Use of the prototype toilet

171 visits to the toilet room were recorded by means of collected survey sheets. Out of those, 167 times the user decided to use the toilet. The users were mostly staff of the PRG laboratories, several of whom used the prototype multiple times. Therefore, the number of individual users was likely much smaller than the recorded number of visits. As can be seen in Table 2, it was used by female and male participants alike, for urination as well as defecation. The question on the type of use (urination and/or defecation) was only introduced to the survey sheets after an initial testing period, hence the smaller usage population. On weekends, the facilities remained closed.

**Table 2:** User statistics of trial at UKZN.

Recorded visits to prototype (by means of collected surveys)	171
Visits on which the user specified they used the Prototype	167 (97.7%)
Male uses	84 (49.1%)
Female uses	85 (49.7%)
User specified their gender as ‘other’	1 (0.6%)
User did prefer not to specify their gender	1 (0.6%)
User specified they only urinated (out of 108 surveys including this question)	63 (58.3%)
User specified they only defecated or defecated and urinated (out of 108 surveys including this question)	44 (40.7%)

#### 3.1.2 Swipe material comparison

As can be seen in Table 3, out of total days tested (d_total_), the toilet bowl was observed clean at the end of the day (d_clean_) more often than not, and without lubrication, the SOB swipe exhibited better performance than the other two materials. Lubrication prior to use improved all swipes' performance, and both silicone swipes performed better than the polyurethane swipe. In total, the bowl was observed to be clean at the end of 30 out of 39 days (77%).

**Table 3:** Days on which the toilet bowl was found clean at the end of the day.

	Polyurethane Swipe (d_clean_/d_total_)	Silicone Swipe (d_clean_/d_total_)	SOB Swipe (d_clean_/d_total_)
No lubrication	4/6 (67%)	4/6 (67%)	5/6 (83%)
With spray lubrication	5/7 (71%)	6/7 (86%)	6/7 (86%)

#### 3.1.3 User surveys

Since some users used the toilet multiple times, they also completed multiple questionnaires. With this pseudo replication, and with a relatively small population size, statistical analysis, e.g. on the impact of gender on a certain user response, was deemed inappropriate. However, the collected answers, (Table 4), can give an indication of the users' likes and dislikes. A three-point Likert-type scale using stylised smiling-, neutral-, and frowning faces was used to represent a positive-, neutral- or undecided-, and negative response, respectively.

a) Odour: The majority of surveys reported no negative odour. It should be noted that in the laboratories outside the toilet test room, analyses of faecal samples were conducted on most days, and this may have influenced the users' ability to detect odour changes inside the toilet room.
b) Cleanliness: Two thirds of user responses reported a clean toilet, while the remaining replies were evenly split between reporting a dirty toilet and an undecided reply.
c) Ease of use: the clearest result was achieved for the question on ease of use, where 87% of submitted responses considered the prototype easy to use and only 2% replied that it was not. It should be noted that the perception of ease of use likely changed over time as participants used the toilet multiple times and became acquainted with it.
d) Toilet preference: The only question that had the highest percentage of replies as “neutral” (68%) was on toilet preference. Of the remaining replies, three times more said to prefer the prototype toilet (24%) than their own (8%).

**Table 4:** User survey responses.

How much do you agree with the statement…	Positive replies	Neutral replies	Negative replies
… “*The toilet didn't smell bad*”? (*n* = 171 responses)	120 (70%)	13 (8%)	38 (22%)
… “*The toilet was clean*”? (n = 171 responses)	113 (66%)	29 (17%)	29 (17%)
… “*The toilet was easy to use*”? (*n* = 169 responses)	147 (87%)	19 (11%)	3 (2%)
… “*I prefer using this toilet to my usual toilet*”? (*n* = 170 responses)	40 (24%)	116 (68%)	14 (8%)

#### 3.1.4 Interviews

Eight users were interviewed about their experience with and attitude towards the prototype. There were four male and four female interviewees, none had a gender expression different from their sex; four were in the age group 30–40 years, three 20–30 years, and one 60–70 years. While the interviewees were from diverse age groups and nationalities, they all had an academic background in sanitation research, and were all accustomed to water flush toilets at home as well as at work.

**Fig. 4:**
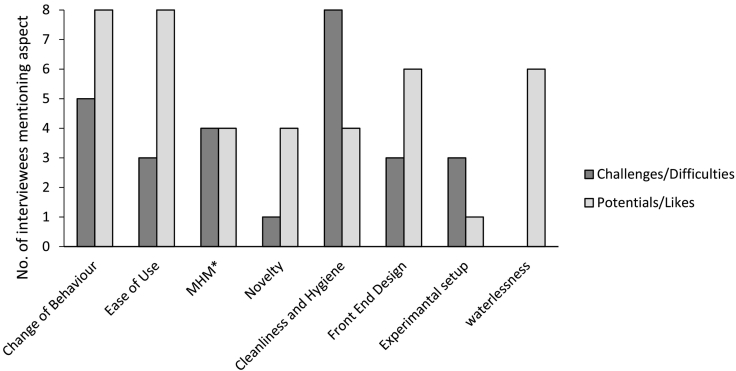
Potential vs Challenges as seen by the 8 interviewees at UKZN. *MHM: Menstrual Hygiene Management – only the four female interviewees were questioned about this aspect.

Most aspects about which interviewees were questioned yielded both positive and negative views (Fig. 4).

Only the four women, believed to be the four interviewees having first-hand experience with menstruation, were questioned about their attitude towards the prototype with regard to menstrual hygiene. All said that they would not mind flushing menstrual absorbents into the toilet if they were permitted to do so. On the other hand, they acknowledged that cleaning menstrual fluid out of the bowl could prove difficult and that blood stains would be of particular concern (compared to faeces). While this was their main concern about using the prototype for menstrual hygiene, they also mentioned the lack of a bin in the toilet room as a barrier.

While some interviewees voiced uncertainty about how the flush mechanism works, a majority said that they had no physical difficulties using the prototype, and that its use was easy to understand. None of them said they had any cultural or religious problems using the prototype: seven out of eight thought if they were to install it in their homes, they would install it in their usual toilet's location, which in the case of all interviewees was inside the house.

All interviewees agreed that they would have liked cleaning equipment to be supplied in the toilet room, or that it would be necessary if they used this pedestal in their homes. Most interviewees mentioned that they thought cleaning would have to be more frequent than, or at least different to, that of a water flush toilet. However, half of the interviewees mentioned that they did not consider cleaning the waterless toilet would be any more difficult or unpleasant than cleaning a water flush toilet.

In six interviews, there was a mention of negative odour being detected at some point during the trial period, but half of all interviewees reported that odour was either neutral or at least tolerable, meaning that odour levels were not exceeding those of a water flush toilet in a similar institutional setting. Two users claimed to have noticed a positive smell (“It smells nice”).

The importance of saving water was discussed in six interviews, and this was the only aspect that was not seen as problematic by any of the interviewees. However, only one interviewee explicitly mentioned they would not mind changing their behaviour for the sake of water preservation.

The novel, clean, design, similar to that of a porcelain toilet was praised, but there was also criticism of the small size and depth of the entire toilet pan, as users disliked the proximity to the excreta.

#### 3.1.5 Additional observations

During the trials, some unexpected issues with the prototype design were discovered. One complaint that was raised by several users, both in person as well as in handwritten comments on the survey sheets, was that they had to aim throwing the toilet paper into the opening of the rotating bowl. If paper fell into any other part of the pan, it would stick there on the damp surface, and the lack of flush water would mean the user would have to remove it manually. One male user complained about having physical contact with the shallow front of the toilet pan when sitting down. Many users mentioned they thought the spray bottle that was provided was helpful, and they suggested incorporating an automated spray into the system, as this could also solve the problem of toilet paper getting stuck. Another observation was a small accumulation of urine residue at the lip of the toilet pan towards the edge of the rotating bowl. This was due to the shape of the pan and should be easily remediated in an updated design.

### 3.2 Household tests

#### 3.2.1 User perception

**Fig. 5:**
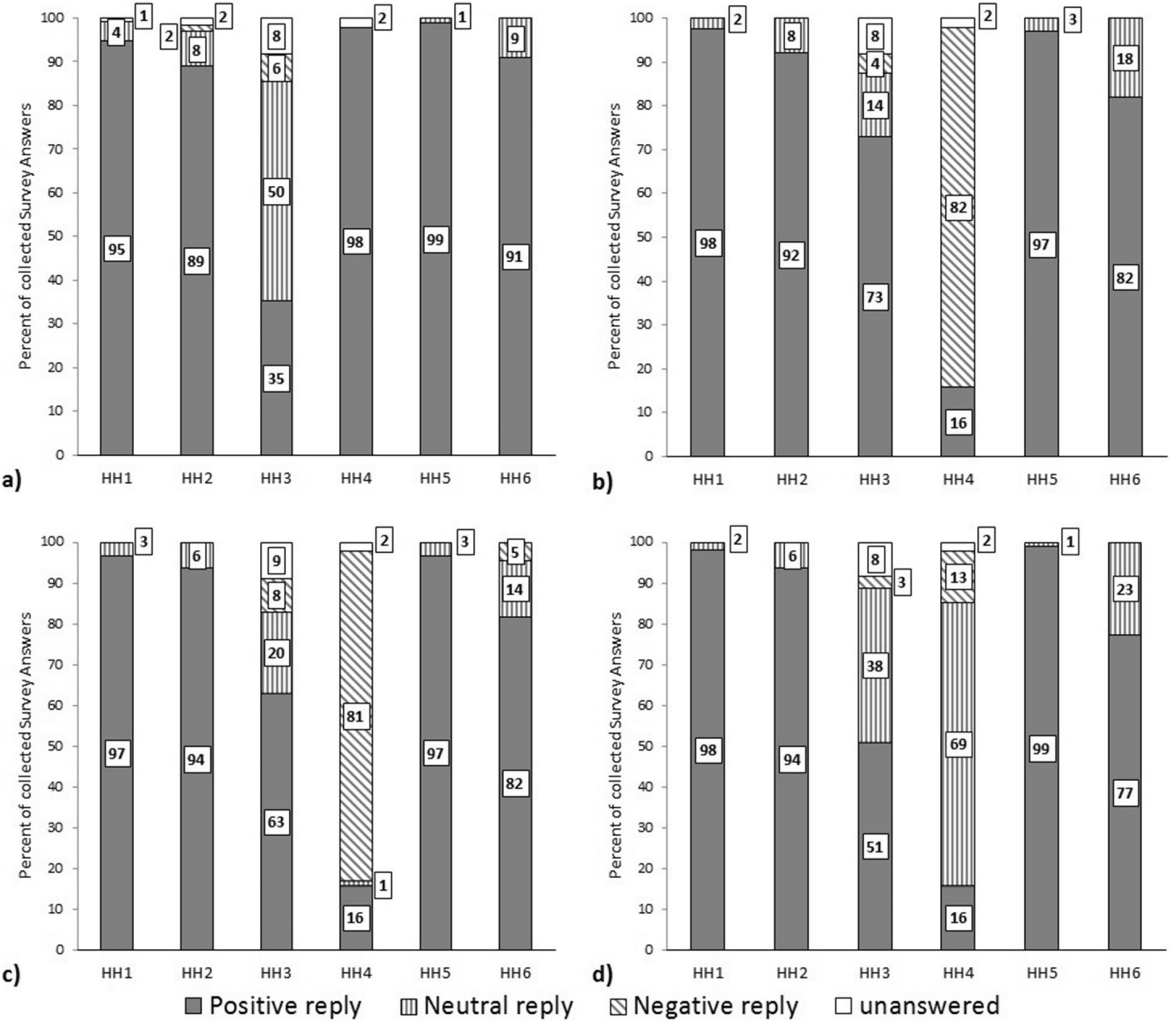
User survey replies from the households, answering the questions “How much do you agree with the statement…?” a) “…the toilet didn't smell bad”, b) “…the toilet was easy to use”, c) “…the toilet was clean”, and d) “…I prefer this toilet to my usual one”.

The two household prototypes were tested in parallel, in three sets of two households. They were well received by four households (HH 1,2,5, and 6 in Table 5 and Fig. 5), whereas the other two households (HH 3 and 4) seemed to be more critical. Most of their user survey replies were more negative or neutral than positive, as compared to households 1,2,5, and 6, whose replies were at least 77% positive for each question (Fig. 5). Surveys were submitted by female and male users as well as users who identified as ‘other’, and a small percentage of users preferred not to disclaim their gender identity (Table 5). Only in the first two households were surveys submitted by people who said they did not use the prototype. Surveys were completed more often by people who had defecated rather than just urinated. Not all surveys were filled out completely (Table 5 and Fig. 5). Whilst households 3 and 4 communicated their reservations, a general trend of positive replies was derived from the user surveys. Of particular interest are the replies to the question about toilet preference. Even household 4, while submitting largely negative replies about their views on ease of use and cleanliness, submitted only a small percentage of replies stating they preferred their usual toilet. Interviews that were conducted with members of each household are currently awaiting publication.

**Table 5:** Demographics of collected user survey replies.

	HH1	HH2	HH3	HH4	HH5	HH6
Gender identity:						
Female (%)	48.8	38.1	46.5	28.4	61.1	50
Male (%)	44.4	54	52.2	25.3	38.9	45.5
Other (%)	0	0	0.6	45.3	0	0
Rather not say (%)	0.5	1.6	0.6	1.1	0	4.5
Left unanswered (%)	6.3	6.3	0	0	0	0

I used the toilet:						
Yes (%)	61.5	87.3	96.9	98.9	93.7	90.9
No (%)	30.2	7.9	0	0	0	0
Left unanswered (%)	8.3	4.8	3.1	1.1	6.3	9.1

I used it for:						
Urinating only (%)	34.1	22.2	27.7	0	10.5	36.4
Defecating (%)	62.4	47.6	64.2	96.8	89.5	59.1
Left unanswered (%)	3.4	30.2	8.2	3.2	0	4.5

#### 3.2.2 Prototype performance

The mechanism in both prototype units functioned without problems in the first set of households (1 and 2) and appeared very clean at almost every visit. However, this was likely impacted because the families used water to keep the toilets clean: During visits water droplets were found on the units' seats and bowls. This was later observed in all households.

In the second set of households, both units started exhibiting signs of fatigue: In household 3, a V-belt ruptured and had to be replaced. The bowl could be observed to have a little fouling when checked during visits. In household 4, the bowl had a failure at the connection point to the gear mechanism. The top half of the unit was replaced with the unit at UKZN, which was out of use by that time.

In the third set of households, while user surveys indicated contentment with the prototypes, water was also used to maintain a cleaner bowl. Both units were observed to have fouling in the bowl during one of the visits, showing that the performance of the swipe and flush mechanism was not always satisfactory. One of the units suffered another failure at the swipe mechanism, which was mitigated by replacing a set of screws.

In all households, blockages occurred of the prototype pedestal's outlet into the vault underneath. This led to material filling up underneath the rotating bowl and eventually impacted the prototype's cleanliness. The blockages could be cleared upon discovery, but might have impacted the users' perception of the prototype.

### 3.3 Swipe test in the laboratory

The removal rates as determined with Eq. (1) for the swipe materials polyurethane, silicone, and SOB, with three different types of faeces, no lubrication, or lubrication with spray and/or synthetic urine (here: SU) are listed in Table 6. With solid faeces (BSC type 3–4), no significant fouling occurred with any swipe material regardless of lubrication. Soft (BSC type 5–6) and liquid (BSC type 7) faeces yielded more diverse results. The error margins indicate a large variation of results among triplicates. Negative values show that, with soft faeces and little to no lubrication, the swipes can smear the fouling over a wider area than originally fouled instead of removing it from the bowl. To compare the swipe materials, all values for the material were averaged, resulting in the last column of Table 6 (Average). The overall swipe efficiency of the SOB swipe material was higher than that of the silicone and polyurethane swipes. The polyurethane swipe achieved the highest removal efficiency for all types of synthetic faeces when spray lubrication and synthetic urine were added. However, without synthetic urine and/or spray, and particularly with soft faeces, the silicone and SOB swipes produce noticeably better results than polyurethane.

**Table 6:** Removal rates determined using ImageJ software.

	Lubricant	Removal rate for solid faeces in %	Removal rate for soft faeces in %	Removal rate for liquid faeces in %	Average for swipe material in %
SOB	Spray + SU	100	64 ± 8	75 ± 6	68 ± 10
	SU	100	36 ± 28	83 ± 9	
	Spray	100	79 ± 10	54 ± 7	
	Dry	100	3 ± 2	18 ± 9	
Silicone	Spray + SU	100	69 ± 29	89 ± 5	62 ± 12
	SU	100	71 ± 17	73 ± 13	
	Spray	100	12 ± 5	25 ± 1	
	Dry	100	−5 ± 1	12 ± 7	
Polyurethane	Spray + SU	100	92 ± 6	90 ± 6	50 ± 17
	SU	100	0 ± 45	52 ± 3	
	Spray	100	−51 ± 7	70 ± 10	
	Dry	100	−53 ± 8	1 ± 7	

Removal rates are average of triplicates; average for swipe material is from all 12 values, error margins are standard error of the mean.

The tests with synthetic menstrual fluid produced no significant results. None of the swipe materials, with or without lubrication, were able to effectively remove blood stains from the rotating bowl's surface. The tampons were successfully flushed out of the bowl, but most staining remained. Therefore, the calculation of removal rates was deemed unnecessary.

## 4 Discussion

The mechanical flush is part of the Nano Membrane Toilet (NMT) project at Cranfield University (Parker, 2014). The NMT is being developed as a standalone, non-sewered, household-level sanitation system that separates solid and liquid wastes (Mercer et al., 2016) and treats them in combustion (Jurado et al., 2018) and membrane processes (Wang et al., 2017) respectively. While a complex technological solution like the NMT could solve a variety of other issues surrounding the storage, transport and treatment of faecal sludge (Strande, 2014), the mechanical flush can be implemented independently, into already existing sanitation systems like pit latrines, chemical toilets, camping, or composting toilets. This could improve their perceived cleanliness, comfort, and hygiene, and thus their acceptance.

The flush's effectiveness in removing faecal matter from the bowl was demonstrated, and the laboratory tests confirmed the preliminary findings regarding the efficiency of swipe materials and use of a lubricating spray. The SOB swipe was least susceptible to fouling, likely due to the oil bleed effect reducing the adhesion of faeces. It also had the best overall removal efficiency, which corresponds to Tierney's (2017) observations about silicone as favourable swipe material. The lubricating spray created a liquid film, which reduced adhesion of faeces to the bowl's surface, thus improving the cleanliness of the prototype. This is one effect of water in water-flush toilets. While a thin film requires far less liquid than even low-volume flush toilets, and such an amount would likely be a valuable use of water for the sake of user acceptance, the idea of an entirely dry flush would be lost were the lubricating spray implemented.

All swipe materials had difficulties removing soft-glutinous synthetic faeces, and the tests using synthetic menstrual fluid highlighted a problem removing blood-stains from the bowl. The fact that the families participating in the household tests used water to keep the prototype clean emphasizes that the dry swipe alone does not yet provide satisfactory cleaning performance. Considering that there is a distinction between actual cleanliness and perceived cleanliness, (Orstad et al., 2017; Vos et al., 2018) a thorough investigation into the stimuli affecting the perception of cleanliness could help focus the development efforts of the mechanical flush. With a combination of design improvements, informed choice of materials, and maintenance protocols, the mechanical flush's cleanliness – and the perception of its cleanliness – could potentially be improved to the level of a porcelain water flush toilet. A customised cleaning utensil for the mechanical flush could facilitate the cleaning process. Fouling in water flush toilets is a common occurrence, both in institutional and household toilets. While the use of toilet brushes is widely accepted in private settings, personal observations would indicate that in public settings, this is not always the case. The authors could not find published studies on the use of toilet brushes, private or institutional. Future user tests of a re-designed prototype could benefit from a parallel investigation of the user habits and cleanliness of institutional water flush toilets.

As an example of the potential for optimisation, the material used for the bowl (polyurethane) was chosen for practical reasons of prototype manufacture and will likely change in the final product, e.g. to reduce the adhesion of faeces. A material or functionalised surface has to be both hydrophobic and oleophobic to fully repel faeces, as they contain both fats and water (Rose et al., 2015). Research on such *omniphobic* surfaces is ongoing (e.g. Wang et al., 2016; Zhu et al., 2017), and could find implementation in the mechanical flush. Such a surface could provide a similar effect as the lubricating spray did, enabling the toilet flush to remain purely mechanical and to not require lubricants.

During household testing, the materials used in the prototype exhibited fatigue, causing failures in moving parts and reduction in swipe efficiency. This is another indicator that further research in material selection is required. The prototype was mainly produced from polyurethane. This is not an unusual choice: portable chemical toilets tend to be made from polyurethane, the UDDT pedestals used in the eThekwini municipality are made from polypropylene (Personal communication with Jacques Rust, Envirosan, Republic of South Africa), and the ‘Blue Diversion Toilet’ developed by EAWAG, 2014, Tobias et al., 2017 is largely made from LLDPE plastic. Given that plastics are cheap, light, durable, and easily available materials, this is unsurprising. Nonetheless, care must be taken to ensure long term durability of all parts in a final product.

The surveys and interviews at UKZN need to be analysed in the knowledge that most users of the prototype, and all interviewees, were members, students, or affiliates of the PRG at UKZN. This means they were well used to working with faecal material and likely perceived sanitation systems differently to the general public. While their opinions are therefore not necessarily generalizable, they likely possessed valuable insights into important aspects of a well-working sanitation technology for use in a water-scarce setting. In addition, these users might be more likely to openly discuss toilet-related matters than the general public. This makes their opinions a useful resource for the next design-iteration of the mechanical toilet flush. Analysing their perception and opinions showed that most users in an institutional setting, who are accustomed to water flush toilets, thought the prototype was easy to use and found odour and cleanliness to be acceptable. However, hygiene, and the process of keeping the toilet bowl clean from both faecal material and menstrual fluid, was a significant concern for these users. The complete lack of water seemed to be problematic to many of them, several of whom suggested installing an automated version of the pump action spray bottle. This highlights the importance of perceived cleanliness (Vos et al., 2018; Whitehead et al., 2007) – it seems that users accustomed to a water flush toilet feel the mechanical flush has a lower level of cleanliness than its aqueous counterpart, even when they are involved in research on dry sanitation technologies. At the same time, the users in households, being accustomed to UDDT-pedestals, perceived the prototype more positively. While flush toilets are still the aspired product for inhabitants of eThekwini municipality (Mkhize et al., 2017), a toilet pedestal designed to look similar to a porcelain toilet and incorporating a waterless mechanical flush seems to be a viable alternative, as long as it is reliably clean and robust.

The swipe tests in the laboratory were a useful simulation of different bodily excreta being flushed, and they confirmed the preliminary findings from the field trials. However, using photography and image analysis to gauge removal rates does not produce perfect results, since the bowl's three-dimensional surface is analysed in a two-dimensional photograph. Photogrammetric methods to develop a three-dimensional image from several two-dimensional photographs of the same object, for example used in medical (Patias, 2002) and forensic research (Urbanová et al., 2015), could be used to deliver more accurate results.

Even though the tests with SMF were inconclusive, they produced the valuable information that the mechanical flush is not yet able to clean blood stains from the toilet bowl. It should, however, be noted that the SMF is only an approximation of real menstrual fluid, and a tampon in real use would not always be as fully soaked as in this experiment (nor would it necessarily be flushed down a toilet). As research into menstrual hygiene management progresses, more accurate methods of simulating menstruation may be developed. Considering the taboos around the subject (Hertz, 2018), the effective removal of blood stains is an important requirement for the acceptance of a mechanical flush.

The mentioned lack of a bin in the toilet room at UKZN likely contributed to the decision of those who menstruate to avoid using the prototype during menses. This was clearly an oversight by the installation team, none of whom menstruate. It indicates a need for greater awareness of menstrual hygiene management issues during preparation of field testing.

### 4.1 Design recommendations

The identified issues with the current prototype design and the users' attitudes translate into several design recommendations:

1. An automated lubricant spray, activated upon opening and/or closing the toilets lid, could improve the cleanliness in the bowl by lubricating the bowls surface prior to use and moving toilet paper from the pan into the bowl. Additionally, this could improve the perception of cleanliness for users accustomed to water flush toilets. This would, however, require a small reservoir of lubricant, and the flush would no longer be completely dry.
2. Deepening the toilet pan/bowl would leave more space between the user and the faeces and reduce the risk of physical contact between the user and the bowl.
3. A change in the pan's geometry would eliminate the issue of urine residue in the pan.
4. Increasing the size of the swipe would increase the contact between swipe and the bowl's surface, and thus the swiping activity.
5. The material for the rotating bowl and the swipe should be resistant to fouling, e.g. with an omniphobic surface, and have a high mechanical strength. If the adhesion of faeces and menstrual fluid is sufficiently low, this could replace the need for the automated lubricant spray.
6. User Guidance on cleaning a waterless toilet could help to ensure that no excess water or inappropriate cleaning chemicals are used.

These recommendations are the result of combined field- and laboratory testing, and will serve as basis for the re-design process of the mechanical flush's next iteration. A re-designed prototype pedestal can then be tested to assess whether the performance and user perception have improved.

Considering the number of design-decisions that have yet to be made in refining the current prototype, for example the selection of materials, the design for manufacture at larger scale, and the location of production, a realistic estimate of the cost of the final product cannot be made at this point.

## 5 Conclusions

In field trials and controlled laboratory experiments, the functionality of a mechanical flush prototype was evaluated.

- It was effective in moving most faecal matter out of the toilet bowl and provided an acceptable odour barrier between faecal material underneath and the environment. Fouling in the bowl was still a persistent problem.
- The laboratory tests gave insight into the selection of swipe material, showing that silicone-based swipes performed better than a polyurethane rubber. They also demonstrated the value of lubricating the bowl before use.
- All swipe materials tested could not sufficiently clean soft-glutinous faeces or blood stains off the bowl's surface, emphasising the difficulty to develop a waterless flush.

Further research should focus on improving the bowl's resistance to fouling as well as the swipe's ability to remove any fouling left in the bowl. Valuable lessons were learned about the potential areas of improvement, and design recommendations were made to inform the next iteration step in developing the mechanical waterless flush. Implemented into other waterless sanitation systems, it could significantly improve their acceptance. Altogether, this study serves as an example of the effective combination of different approaches to increase the insight gained from testing a prototype.

## Declarations of interest

None.

## Figures and Tables

**Fig. A.1:**
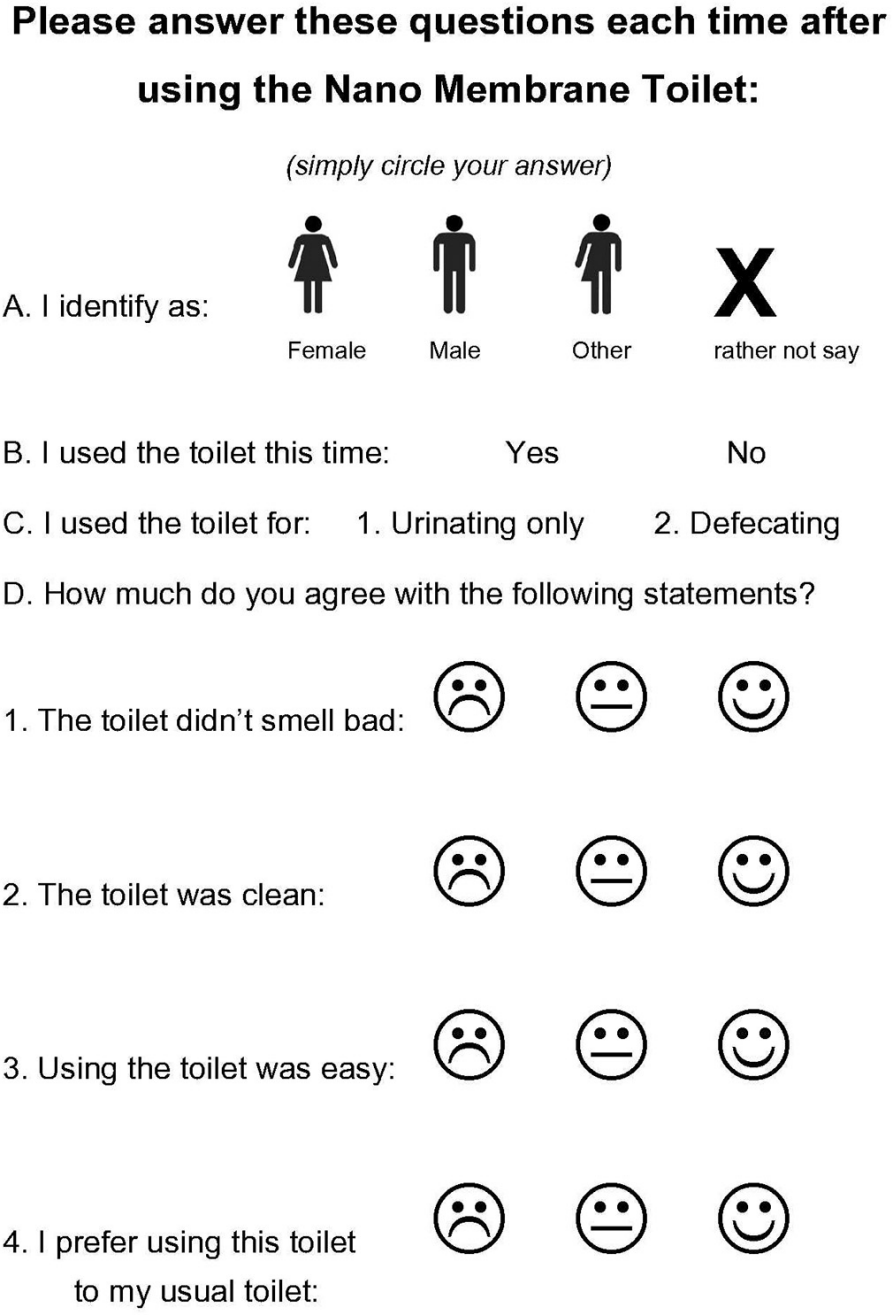
Survey questionnaire for user testing at UKZN.

**Table B.1:** Topics covered in the semi-structured interviews with users of the prototype at UKZN.

Likes and dislikes about the toilet
Smell
Cleanliness
Challenges using the toilet
Difficulties Cleaning the toilet
Menstrual hygiene management (MHM; was only discussed with female interviewees)
Comparison to usual toilet
Toilet location
Cultural and religious norms
Overall experience

**Fig. C.1:**
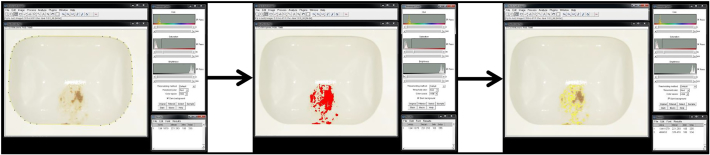
Using ImageJ Software to determine covered area in toilet bowl.

**Table D.1:** Simplified recipe for 200 g synthetic faeces.

40 g dry brewer's yeast
20 g psyllium husks
5 g plain white wheat flour
15 g Miso paste
15 g Polyethylene glycol (PEG) 4000
15 g CaPO_3_
10 g cellulose powder
80 g deionised water
First, all dry ingredients are mixed together, then the Miso paste and water are added. Everything is stirred thoroughly.

**Fig. E.1:**
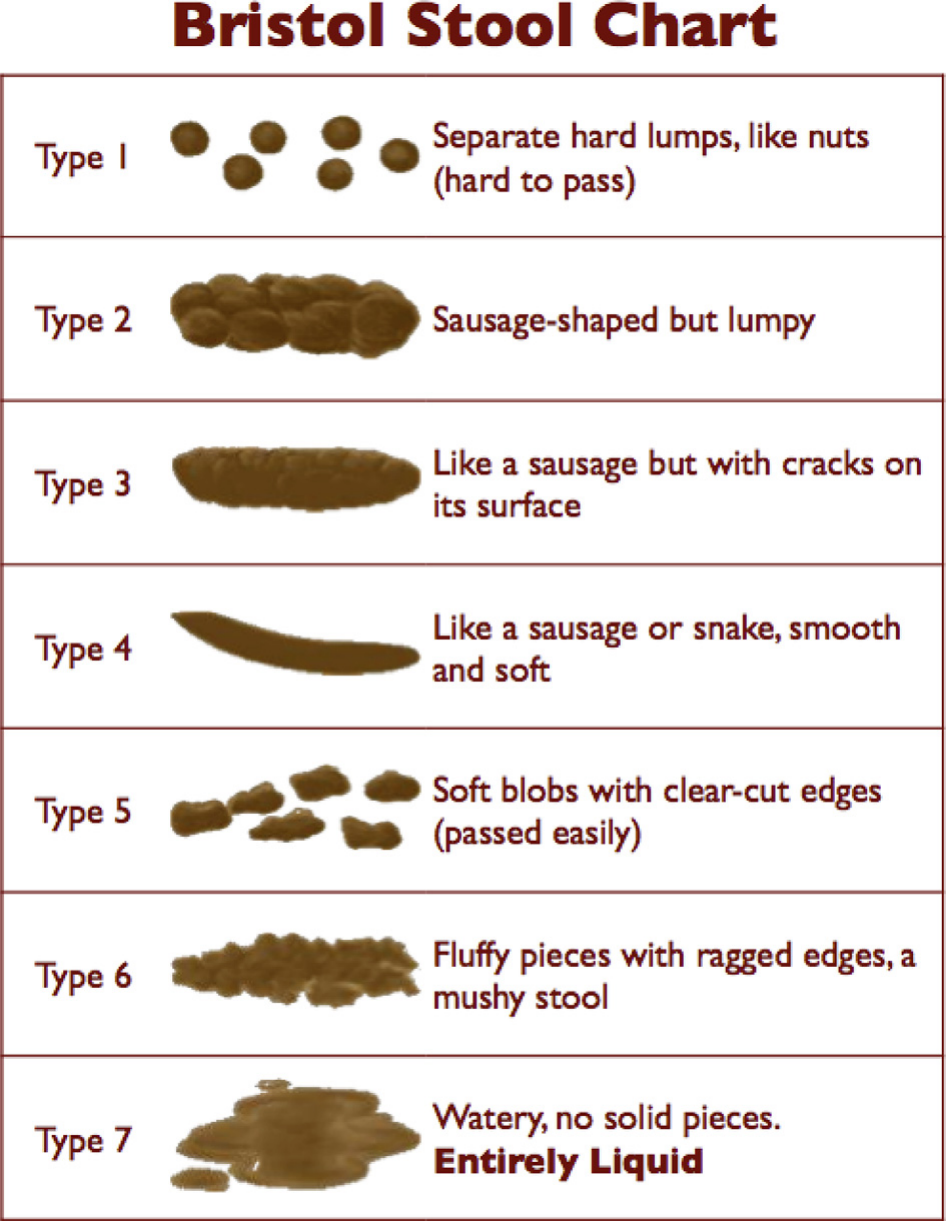
Bristol stool chart; image created by Kyle Thompson for Michigan Medicine, University of Michigan, under a Creative Commons Attribution-NonCommercial-ShareAlike 3.0 Unported License.

## Appendix C Procedure for determining fouling removal rate

In *ImageJ* software, the area of the rotating bowl was determined in each picture by tracing its outline, using the *polygon selection tool* and measuring the area within in pixels. Then, with the *Color threshold* tool, the area covered in synthetic faeces was determined, using the following settings:

• *Hue* and *Brightness*: set to full range
• *Saturation*: from 50 to 255.

These settings were found to capture the faeces in the image, but not the white bowl, or shadows therein. Every photograph had to be analysed individually, because the zoom changed slightly between photographs. Figure demonstrates the process.

The covered areas before and after flushing (measured in pixels) could be compared as ratios to the area of the bowl. The removal rate was calculated from the ratio of these ratios before to after flushing, as seen in Eq. (1).

(C.1) $$\text{removal rate}=1-\frac{{\left(\frac{A_{\text{covered}}}{A_{\text{bowl}}}\right)}_{\text{After Flush}}}{{\left(\frac{A_{\text{covered}}}{A_{\text{bowl}}}\right)}_{\text{Before Flush}}}$$

## Appendix D Recipe for synthetic faeces

To increase adhesion of residue on the toilet bowl's surface, peanut oil was removed from the original recipe and plain white wheat flour was added. In the quantities used for this study (200 g synthetic faeces at a time), it was feasible to simplify the portions of all ingredients by rounding to the closest multiple of 5 g:
